# Population attributable fraction of all-cause mortality due to non-normal blood pressure: results from the 2013 to 2016 baseline survey of the TMM CommCohort Study

**DOI:** 10.1038/s41440-025-02436-0

**Published:** 2025-11-12

**Authors:** Rieko Hatanaka, Naoki Nakaya, Mana Kogure, Kumi Nakaya, Ippei Chiba, Sayuri Tokioka, Masato Takase, Yuka Kotozaki, Taku Obara, Satoshi Nagaie, Hideki Ohmomo, Takahito Nasu, Michihiro Satoh, Takahisa Murakami, Hirohito Metoki, Yohei Hamanaka, Masatsugu Orui, Eiichi N. Kodama, Nobuo Fuse, Yoko Izumi, Kozo Tanno, Atsushi Hozawa

**Affiliations:** 1 Tohoku Medical Megabank Organization, Tohoku University, Sendai, Miyagi Japan; 2 Graduate School of Medicine, Tohoku University, Sendai, Miyagi Japan; 3 Iwate Tohoku Medical Megabank Organization, Iwate Medical University, Shiwa-gun, Iwate Japan; 4 Tohoku University Hospital, Tohoku University, Sendai, Miyagi Japan; 5 Tohoku Medical and Pharmaceutical University, Sendai, Japan; 6 International Research Institute of Disaster Science, Tohoku University, Sendai, Miyagi Japan

**Keywords:** Population attributable fraction, All-cause mortality, Blood pressure, Hypertension

## Abstract

Antihypertensive therapy has reduced cardiovascular mortality; however, challenges remain, including residual risk in treated patients and the population burden associated with borderline hypertension. Previous Japanese estimates of the population attributable fraction (PAF) are derived from older cohorts and often lacked stratification by treatment status. We conducted a prospective study of 61,495 participants (women: 56.7%, aged 60.7 ± 11.0 years) from the Tohoku Medical Megabank Community-Based Cohort Study. Participants were classified into six blood pressure (BP) categories based on the JSH 2019 guidelines, and further stratified by hypertension treatment status, resulting in 12 groups. Using untreated individuals with normal BP as the reference, we calculated multivariable-adjusted hazard ratios (HR), 95% confidence intervals (CI), and PAF for the remaining groups using Cox proportional hazards model. During a median follow-up of 6.5 years, 1909 deaths were recorded. HRs increased with rising BP in both untreated and treated participants. The overall PAF for all-cause mortality due to non-normal BP was 9.45%, with a marked sex difference (12.25% in male and 5.16% in female). The highest PAF contributions were observed in the treated Grade I hypertension group (2.18%) and the untreated elevated BP group (1.28%). In this contemporary Japanese cohort, non-normal BP accounts for 9.45% of all-cause mortality, representing a substantial public health burden, particularly among men. The substantial PAF contributions from both treated patients and untreated individuals with elevated BP highlight the importance of effective BP management for both primary and secondary prevention.

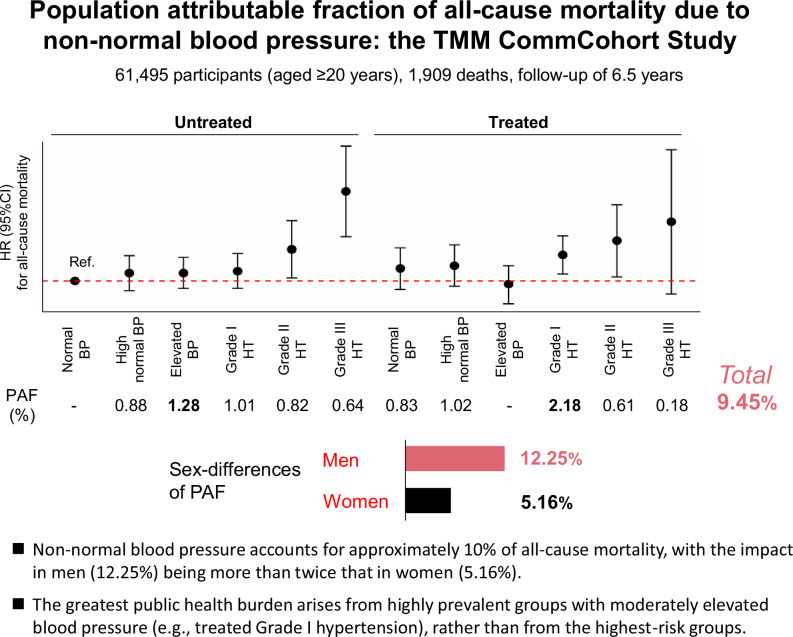

## Introduction

Blood pressure (BP) lowering therapy is a cornerstone of modern cardiovascular disease prevention, and its widespread adoption has substantially reduced mortality from cardiovascular diseases (CVD), including stroke and myocardial infarction [1–3]. Despite these advances, significant clinical challenges remain. One such factor is the residual risk observed even in patients who achieve target BP levels under treatment [4, 5]. Furthermore, untreated borderline conditions, such as prehypertension and elevated BP, which are not typically targeted for pharmacological intervention but are highly prevalent, represent a major contributor to the overall burden of CVD in the population [6].

Most existing research has focused on assessing relative risk at the individual level, whereas large-scale studies quantifying the population attributable fraction (PAF) of non-normal BP for all-cause mortality in contemporary Japan remain limited [7, 8]. PAF is a public health indicator that estimates the proportion of a disease burden across an entire population attributable to a specific risk factor.

Therefore, this study aimed to quantitatively assess the impact of non-normal BP on all-cause mortality using PAF, based on recent guidelines, and leveraging large-scale community-based cohort data from the Tohoku Medical Megabank (TMM) Project. Particular emphasis was placed on strict stratification of participants according to antihypertensive treatment status to enable a more precise risk assessment.

Point of view

- Clinical relevance
  The results of this study provide important clinical implications for Japanese clinicians, stating that the contribution of moderate-risk patients, such as those with “high blood pressure” that does not reach the threshold for drug treatment and those with “stage 1 hypertension” that is within the management target, to mortality should not be overlooked. Early lifestyle advice and more active follow-up for these patients are warranted.
- Future direction
  The contribution of non-normal blood pressure revealed in this study needs to be broken down and analyzed by cause of death (e.g., stroke, heart disease, renal failure, etc.) to identify the main pathological conditions leading to death.
- Consideration for the Asian population
  The quantified mortality burden from hypertension in Japan, the world’s first super-aged society, serves as an important leading indicator of the magnitude of the public health challenges that other Asian countries, which are also facing rapid aging and urbanization, will face in the future.

## Methods

### Study setting, design

The TMM Project was conducted jointly by the Tohoku University Tohoku Medical Megabank Organization (ToMMo) and Iwate Medical University Iwate Tohoku Medical Megabank Organization (IMM) [9]. The TMM Project includes two prospective cohort studies in Miyagi and Iwate: the TMM Community-Based Cohort Study (TMM CommCohort Study), which is a population-based adult cohort study [10, 11] and the TMM Birth and Three-Generation Cohort Study (TMM BirThree Cohort Study), which is a birth and three-generation cohort study [12].

The baseline survey for the TMM CommCohort Study was conducted between May 2013 and March 2016 among residents aged ≥20 years living in Miyagi and Iwate, through two approaches. Type 1 surveys were administered at specific municipal health check-ups, while Type 2 survey was conducted at the Community Support Center in ToMMo and IMM. In both surveys, blood and urine tests were collected, and self-administered questionnaires were obtained. This prospective study utilized data exclusively from the Type 1 survey.

### Ethical considerations

The study protocol conformed to the ethical guidelines of the 1975 Declaration of Helsinki, and was reviewed and approved by the Ethics Committee of ToMMo, Tohoku University (first edition: 2012-4-617, latest edition: 2024-4-049). At the IMM, approval from the Ethics Committee of Iwate Medical University was obtained on April 4, 2013 (HGH25-2). The latest approval (revised 17th edition) was obtained on December 14, 2023. Written informed consent was obtained from each participant before participation in the study.

### BP classification

Each participant was measured systolic BP (SBP) and diastolic BP (DBP) in a sitting position using an upper arm automatic sphygmomanometer at the site of the specific municipal health checkup. BP was measured twice in accordance with the instructions of the Ministry of Health, Labor, and Welfare. However, depending on the situation at the municipalities’ health check-up sites, one measurement was allowed. The first value was used in the analysis.

Based on JSH2019 [13], each participant was classified into six categories: Normal BP, SBP < 120 mmHg and DBP < 80 mmHg; High normal BP, 120 ≤ SBP ≤ 129 mmHg and DBP < 80 mmHg; Elevated BP, 130 ≤ SBP ≤ 139 mmHg and/or 80 ≤ DBP ≤ 89 mmHg; Grade I hypertension (HT), 140 ≤ SBP ≤ 159 mmHg and/or 90 ≤ DBP ≤ 99 mmHg; Grade II HT, 160 ≤ SBP ≤ 179 mmHg and/or 100 ≤ DBP ≤ 109 mmHg; and Grade III HT, 180 mmHg ≤ SBP and/or 110 mmHg ≤ DBP. The JSH 2019 classification was originally designed for individuals not receiving antihypertensive treatment; however, in this study, it was applied to all participants, including those undergoing treatment, for consistency. Additionally, “non-normal BP” was defined as either having untreated high-normal BP or higher, or receiving antihypertensive treatment.

### Treatment status for hypertension

Hypertension treatment status was assessed at baseline using a self-administered questionnaire. Participants who responded “I am currently under treatment,” were classified as the treatment group; all others were classified under untreated group. Participants who did not respond to the hypertension treatment status question were also classified as untreated.

### Covariates

A self-administered questionnaire was used to collect information on smoking status, alcohol status, educational attainment, marital status, physical activity, depressive symptoms, diabetes treatment status, dyslipidemia treatment status, history of CVD (cerebral hemorrhage, cerebral infarction, subarachnoid hemorrhage, myocardial infarction, and angina pectoris), and history of cancer.

Smoking status was classified as current, ex-, or never-smoker. Participants who reported smoking fewer than 100 cigarettes in their lifetime were classified as never smokers. Those who smoked 100 or more cigarettes but were not currently smoking were classified as ex-smokers. Participants who had smoked 100 or more cigarettes and continued to smoke were classified current smokers.

Drinking status was classified into three categories: current, ex-, and never-drinkers. Participants were classified as current drinkers if they answered ‘yes’ to the question ‘Do you drink alcohol?’, ex-drinkers if they answered “quit”, and never drinkers if they answered “rarely (never) drink” or “constitutionally unable to drink.” Metabolic equivalent (MET) hours/day was calculated by multiplying the MET score for a specific activity by the hours per day spent on that activity [14]. Participants were then grouped into quartiles (Q1–Q4) based on their MET・hours per day.

Depressive symptoms were assessed using a self-administered Center for Epidemiologic Studies Depression Scale (CES-D) questionnaire [15, 16]. A CES-D score of ≥ 16 was defied as indicative of depression symptom [16]. Missing data were categorized as unknown.

Estimated 24-h sodium and potassium excretion levels were calculated using Tanaka’s formula [17]. The urinary sodium-to-potassium (Na/K) ratio was calculated by dividing the estimated 24-h sodium excretion by the potassium excretion. Body mass index (BMI) was calculated as weight (kg) divided by height squared (m). Height and weight were measured at a specific municipal health checkup site.

### Confirmation of all-cause mortality

ToMMo and IMM employed different methods for participant follow-up. ToMMo reviewed the municipal basic resident register almost annually to identify participants who met any of the following categories: 1) death was reported by family members, 2) the address was unknown and mail could not be delivered, and 3) withdrawal from the National Health Insurance was confirmed due to death. In contrast, IMM identified deaths and relocations by annually collating municipal basic resident register data electronically or by requesting a certificate of residence from all participants.

‘Death’ was defined from the date of death. Participants who relocated and could not be followed for vital status were censored on the date of relocation. For participants without these events, the follow-up period extended to the last day of follow-up. The follow-up period for the survival analysis ended on December 31, 2021. Withdrawal of consent was monitored using data available up to December 11, 2023.

### Statistical analyses

The basic characteristics of participants were summarized by BP category according to the JSH2019 classification, separately for treated and untreated participants. Cox proportional hazard model was used to calculate the multivariable adjusted hazard ratio (HR) and 95% confidence intervals (CI) for all-cause mortality by BP classification in both groups. Covariates used in the model comprised age in years (20–39, 40–49, 50–59, 60–69, ≥70), sex, study area (Miyagi, Iwate), fiscal year of survey participation (FY2013, FY2014, FY2015), season of the survey (spring or autumn, summer or winter), BMI (<18.5 kg/m^2^, 18.5–24.9 kg/m^2^, ≥25.0 kg/m^2^), smoking status (current, ex-, never-smoker, unknown), drinking status (current, ex-, never-drinker, unknown), educational attainment (elementary/junior high/high school, vocational school/junior college/technical college, university or higher, other, unknown), marital status (married, unmarried, separated, widowed, unknown), physical activity (Q1–Q4, unknown), depressive symptom (presence, absence, unknown), treatment of diabetes (presence, absence, unknown), treatment of dyslipidemia (presence, absence, unknown), history of cardiovascular disease (presence, absence), history of cancers (presence, absence), and Na/K ratio. The analyses were conducted according to sex.

We further calculated the PAF as pd × {HR-1}/HR, where pd is the proportion of cases exposed to the risk factor [18]. PAF was calculated for each group except for the normal BP group. Additionally, the sum of the PAFs calculated for each group was used to determine the overall PAF of all-cause mortality attributable to the non-normal BP.

In addition, two sensitivity analyses were performed. First, to align with previous studies [7, 8], the analysis was restricted to participants aged ≥40 years with no history of CVD. Second, participants who did not respond to the question regarding hypertension treatment status were excluded.

All statistical analyses were performed using SAS version 9.4 (SAS Inc., Cary, NC, USA). Two-tailed *p*-values < 0.05 were considered statistically significant.

## Results

**Fig. 1:**
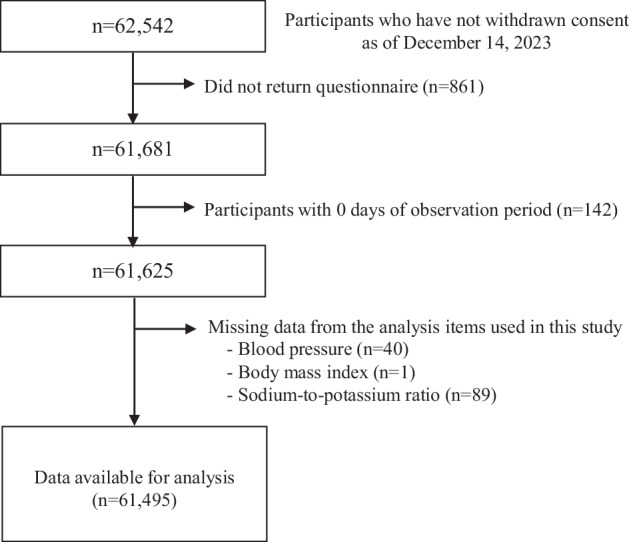
Flowchart of study participants

There were 62,542 participants who were enrolled in the baseline survey and had not withdrawn their consent as of December 2023 (Fig. 1). The following participants were excluded: those who did not return the questionnaire (*n* = 861); those who had an observation period of 0 days (*n* = 142); those with missing BP data (*n* = 40); those with missing BMI data (*n* = 1), and those with missing sodium-to-potassium ratio data (*n* = 89). Ultimately, 61,495 participants were included in the analysis. The total observation period was 401,902 person-years, with 1909 deaths.

The basic characteristics of untreated and treated participants according to BP classification are shown in Table 1. Among untreated participants, those with higher BP tended to be older, male, obese with a BMI of ≥25 kg/m^2^, current smoker, current drinker, physically active, exhibit high Na/K ratio, and low rates of depression. Among treated participants, higher BP was associated with greater proportion of obesity, higher physical activity, higher prevalence of dyslipidemia treatment, elevated Na/K ratio, and lower prevalence of depression.

**Table 1:** Basic characteristics of the untreated and treated participants according to blood pressure classification

	Untreated												Treated											
	Normal BP		High normal BP		Elevated BP		Grade I HT		Grade II HT		Grade III HT		Normal BP		High normal BP		Elevated BP		Grade I HT		Grade II HT		Grade III HT	
	(*n* = 17,458)		(*n* = 8085)		(*n* = 11,176)		(*n* = 6425)		(*n* = 1471)		(*n* = 299)		(*n* = 3158)		(*n* = 3113)		(*n* = 5532)		(*n* = 3923)		(*n* = 704)		(*n* = 151)	
Age, years																								
20–39	2864	(16.4)	621	(7.7)	575	(5.1)	160	(2.5)	36	(2.5)	4	(1.3)	4	(0.1)	7	(0.2)	12	(0.2)	12	(0.3)	5	(0.7)	0	(0.0)
40–49	2790	(16.0)	847	(10.5)	1140	(10.2)	469	(7.3)	132	(9.0)	29	(9.7)	63	(2.0)	34	(1.1)	140	(2.5)	63	(1.6)	13	(1.9)	7	(4.6)
50–59	3203	(18.4)	1392	(17.2)	2151	(19.3)	1160	(18.1)	273	(18.6)	66	(22.1)	296	(9.4)	247	(7.9)	661	(12.0)	390	(9.9)	78	(11.1)	20	(13.3)
60–69	6346	(36.4)	3630	(44.9)	5303	(47.5)	3192	(49.7)	727	(49.4)	142	(47.5)	1737	(55.0)	1724	(55.4)	3028	(54.7)	2090	(53.2)	391	(55.5)	80	(53.0)
≥70	2255	(12.9)	1595	(19.7)	2007	(18.0)	1444	(22.5)	303	(20.6)	58	(19.4)	1058	(33.5)	1101	(35.4)	1691	(30.6)	1368	(34.9)	217	(30.8)	44	(29.1)
Sex																								
Female	12,808	(73.4)	5501	(68.0)	6432	(57.6)	3653	(56.9)	775	(52.7)	136	(45.5)	1722	(54.5)	1779	(57.2)	2913	(52.7)	2071	(52.8)	370	(52.6)	92	(60.9)
Study area																								
Miyagi	10,583	(60.6)	4267	(52.8)	7059	(63.2)	3719	(57.9)	954	(64.9)	199	(66.6)	1655	(52.4)	1438	(46.2)	3438	(62.2)	2168	(55.3)	441	(62.6)	93	(61.6)
Iwate	6875	(39.4)	3818	(47.2)	4117	(36.8)	2706	(42.1)	517	(35.2)	100	(33.4)	1503	(47.6)	1675	(53.8)	2094	(37.9)	1755	(44.7)	263	(37.4)	58	(38.4)
Fiscal year of survey participation																								
FY2013	4135	(23.7)	2109	(26.1)	2837	(25.4)	1767	(27.5)	407	(27.7)	81	(27.1)	673	(21.3)	819	(26.3)	1384	(25.0)	1048	(26.7)	194	(27.6)	42	(27.8)
FY2014	7146	(40.9)	3248	(40.2)	4573	(40.9)	2514	(39.1)	537	(36.5)	115	(38.5)	1343	(42.5)	1269	(40.8)	2300	(41.6)	1510	(38.5)	248	(35.2)	59	(39.1)
FY2015	6177	(35.4)	2728	(33.7)	3766	(33.7)	2144	(33.4)	527	(35.8)	103	(34.5)	1142	(36.2)	1025	(32.9)	1848	(33.4)	1365	(34.8)	262	(37.2)	50	(33.1)
Season of the survey																								
Spring or autumn	7528	(43.1)	3786	(46.8)	4972	(44.5)	3153	(49.1)	724	(49.2)	149	(49.8)	1277	(40.4)	1405	(45.1)	2410	(43.6)	1950	(49.7)	389	(55.3)	92	(60.9)
Summer	9221	(52.8)	3876	(47.9)	5661	(50.7)	2911	(45.3)	652	(44.3)	126	(42.2)	1753	(55.5)	1542	(49.5)	2892	(52.3)	1779	(45.4)	285	(40.5)	49	(32.5)
Winter	709	(4.1)	423	(5.2)	543	(4.9)	361	(5.6)	95	(6.7)	24	(8.0)	128	(4.1)	166	(5.3)	230	(4.2)	194	(5.0)	30	(4.3)	10	(6.6)
BMI, kg/m^2^																								
≥25.0	2671	(15.3)	1996	(24.7)	3444	(30.8)	2245	(34.9)	562	(38.2)	139	(46.5)	1272	(40.3)	1340	(43.1)	2581	(46.7)	1876	(47.8)	365	(51.9)	79	(52.3)
SBP, mmHg	108.0	(7.9)	124.2	(2.9)	130.6	(6.6)	146.1	(7.3)	163.2	(8.7)	183.3	(15.8)	111.1	(6.6)	124.6	(2.9)	131.6	(6.0)	146.7	(6.9)	164.5	(7.5)	185.6	(15.0)
DBP, mmHg	66.2	(6.8)	72.2	(5.0)	80.2	(5.4)	85.7	(7.0)	94.6	(8.7)	105.3	(12.3)	68.4	(6.4)	72.0	(5.1)	79.5	(5.9)	84.1	(7.1)	91.1	(9.7)	100.8	(12.8)
Smoking status																								
Current smoker	2637	(15.1)	1058	(13.1)	1724	(15.4)	892	(13.9)	236	(16.0)	65	(21.7)	416	(13.2)	345	(11.1)	621	(11.2)	421	(10.7)	66	(9.4)	15	(9.9)
Drinking status																								
Current drinker	7828	(44.9)	3615	(44.7)	5676	(50.8)	3302	(51.4)	831	(56.5)	187	(62.5)	1572	(49.8)	1536	(49.3)	2984	(53.9)	2055	(52.4)	373	(53.0)	80	(53.0)
Educational background																								
University or higher	1588	(9.1)	596	(7.4)	867	(7.8)	420	(6.5)	125	(8.5)	27	(9.0)	210	(6.7)	178	(5.7)	377	(6.8)	232	(5.9)	38	(5.4)	8	(5.3)
Marital status																								
Married	13,178	(75.5)	6172	(76.3)	8464	(75.7)	4929	(76.7)	1123	(76.3)	209	(69.9)	2519	(79.8)	2497	(80.2)	4482	(81.0)	3197	(81.5)	542	(77.0)	122	(80.8)
Physical activity, METs (hour/day)																								
75% quartile range or higher	3466	(19.9)	1910	(23.6)	2738	(24.5)	1578	(24.6)	352	(23.9)	74	(24.8)	753	(23.8)	760	(24.4)	1332	(24.1)	1009	(25.7)	166	(23.6)	44	(29.1)
Depressive symptom																								
Presense	4877	(27.9)	2001	(24.8)	2687	(24.0)	1398	(21.8)	308	(20.9)	64	(21.4)	856	(27.1)	791	(25.4)	1280	(23.1)	869	(22.2)	161	(22.9)	33	(21.9)
Diabetes treatment status																								
Presence	572	(3.3)	358	(4.4)	467	(4.2)	266	(4.1)	46	(3.1)	7	(2.3)	419	(13.3)	470	(15.1)	711	(12.9)	549	(14.0)	116	(16.5)	20	(13.3)
Dyslipidemia treatment status																								
Presence	1157	(6.6)	670	(8.3)	909	(8.1)	458	(7.1)	70	(4.8)	9	(3.0)	765	(24.2)	747	(24.0)	1283	(23.2)	829	(21.1)	149	(21.2)	22	(14.6)
Histroy of cadiovascular disease																								
Presense	459	(2.6)	240	(3.0)	358	(3.2)	197	(3.1)	43	(2.9)	6	(2.0)	406	(12.9)	322	(10.3)	545	(9.9)	363	(9.3)	74	(10.5)	10	(6.6)
Histroy of cancer																								
Presense	1169	(6.7)	555	(6.9)	755	(6.8)	438	(6.8)	110	(7.5)	15	(5.0)	314	(9.9)	282	(9.1)	503	(9.1)	311	(7.9)	53	(7.5)	9	(6.0)
Na/K ratio	4.0	(1.0)	4.0	(0.9)	4.1	(1.0)	4.2	(1.0)	4.3	(1.0)	4.4	(1.1)	3.9	(1.0)	4.0	(1.0)	4.1	(1.0)	4.2	(1.0)	4.4	(1.0)	4.4	(1.1)

*BMI* body mass index, *BP* blood pressure, *DBP* diastolic blood pressure, *HT* Hypertension, *Na/K ratio* Sodium-to-potassium ratio, *SBP* systolic blood pressure

The values in the table are presented as means (standard deviations) for continuous variables and as numbers (percentage) for categorical variables

Table 2 presents the multivariable adjusted HR for all-cause mortality according to BP classification and hypertension treatment status. Using untreated normal BP participants as the reference group, HRs increased progressively with higher BP for both untreated and treated participants. The PAF for all-cause mortality in participants with non-normal BP was 9.45%. Among all groups, the highest PAF was observed in the treated Grade I group (2.18%), followed by the untreated elevated BP group (1.28%).

**Table 2:** Multivariable adjusted hazard ratios of all-cause mortality according to BP classification, hypertension treatment status, and PAF

	No. of death	No. of participants	Total person-years	HR (95% CI)^a^	HR (95% CI)^b^	HR (95% CI)^c^	PAF (%)
Untreated							
Normal BP	364	17,458	114,251	1.00 (Ref.)	1.00 (Ref.)	1.00 (Ref.)	–
High normal BP	227	8085	53,426	1.04 (0.88–1.23)	1.07 (0.91–1.27)	1.08 (0.91–1.28)	0.88
Elevated BP	329	11,176	72,599	1.03 (0.89–1.20)	1.07 (0.92–1.25)	1.08 (0.93–1.26)	1.28
Grade I HT	212	6425	42,066	1.03 (0.87–1.22)	1.09 (0.92–1.30)	1.10 (0.93–1.31)	1.01
Grade II HT	59	1471	9440	1.30 (0.99–1.71)	1.36 (1.03–1.80)	1.36 (1.03–1.80)	0.82
Grade III HT	21	299	1866	2.28 (1.46–3.54)	2.38 (1.53–3.71)	2.39 (1.54–3.72)	0.64
Treated							
Normal BP	137	3158	20,683	1.14 (0.93–1.39)	1.12 (0.91–1.37)	1.13 (0.92–1.38)	0.83
High normal BP	141	3113	20,715	1.14 (0.94–1.39)	1.14 (0.93–1.40)	1.16 (0.95–1.42)	1.02
Elevated BP	190	5532	35,971	0.93 (0.78–1.11)	0.96 (0.79–1.15)	0.97 (0.80–1.16)	-
Grade I HT	185	3923	25,439	1.22 (1.02–1.46)	1.25 (1.04–1.51)	1.29 (1.07–1.55)	2.18
Grade II HT	36	704	4475	1.43 (1.02–2.02)	1.48 (1.04–2.10)	1.48 (1.04–2.10)	0.61
Grade III HT	8	151	971	1.65 (0.82–3.32)	1.70 (0.84–3.43)	1.78 (0.88-3.59)	0.18
Total	1909	61,495	401,902				9.45

PAFs with HR less than 1 are displayed as “–”

Normal BP, SBP < 120 mmHg and DBP < 80 mmHg; High normal BP, 120 ≤ SBP ≤ 129 mmHg and DBP < 80 mmHg; Elevated BP, 130 ≤ SBP ≤ 139 mmHg and/or 80 ≤ DBP ≤ 89 mmHg

Grade I HT, 140 ≤ SBP ≤ 159 mmHg and/or 90 ≤ DBP ≤ 99 mmHg; Grade II HT, 160 ≤ SBP ≤ 179 mmHg and/or 100 ≤ DBP ≤ 109 mmHg; and Grade III HT, 180 mmHg ≤ SBP and/or 110 mmHg ≤ DBP

*BP* blood pressure, *CI* confidence interval, *HR* hazard ratio, *HT* hypertension, *PAF* population-attributable fraction

^a^Adjusted for age, sex, study area, fiscal year of survey participation, season of the survey

^b^Adjusted for age, sex, study area, fiscal year of survey participation, season of the survey, smoking status, drinking status, BMI, diabetes treatment, dyslipidemia treatment, history of cardiovascular disease, sodium-to-potassium ratio

^c^Adjusted for age, sex, study area, fiscal year of survey participation, season of the survey, smoking status, drinking status, BMI, diabetes treatment, dyslipidemia treatment, history of cardiovascular disease, sodium-to-potassium ratio, educational attainment, marital status, physical activity, depressive symptom, history of cancers

Table 3 presents the multivariable adjusted HR for all-cause mortality according to BP classification and hypertension treatment status, stratified by sex. Among males, higher BP was associated with an increased risk of all-cause mortality in both untreated and treated groups, except for treated Grade III. Similarly, among females, higher BP was associated with a higher risk for all-cause mortality in both untreated and treated participants. The PAF for all-cause mortality associated with non-normal BP was 12.25% in male and 5.16% in female.

**Table 3:** Multivariable adjusted hazard ratios of all-cause mortality according to BP classification, hypertension treatment status, and PAF, stratified by sex

	No. of death	No. of participants	Total person-years	Hazard ratio (95% CI)^a^	PAF(%)
Men					
Untreated					
Normal BP	198	4650	30,027	1.00 (Ref.)	–
High normal BP	139	2584	16,747	1.16 (0.93–1.44)	1.54
Elevated BP	224	4744	30,427	1.13 (0.93–1.37)	2.07
Grade I HT	144	2772	17,964	1.14 (0.92–1.42)	1.42
Grade II HT	40	696	4436	1.34 (0.95–1.89)	0.82
Grade III HT	16	163	997	2.50 (1.50–4.18)	0.77
Treated					
Normal BP	95	1436	9288	1.16 (0.90–1.49)	1.05
High normal BP	89	1334	8749	1.11 (0.86–1.44)	0.71
Elevated BP	134	2619	16,867	0.995 (0.79–1.25)	–
Grade I HT	138	1852	11,817	1.40 (1.12–1.77)	3.17
Grade II HT	25	334	2081	1.51 (0.99–2.31)	0.68
Grade III HT	3	59	377	1.14 (0.36–3.58)	0.03
Total	1,245	23,243	149,777		12.25

	No. of death	No. of participants	Total person-years	Hazard ratio (95% CI)^a^	PAF(%)
Women					
Untreated					
Normal BP	166	12,808	84,224	1.00 (Ref.)	-
High normal BP	88	5501	36,679	0.96 (0.74–1.24)	–
Elevated BP	105	6432	42,172	0.995 (0.78–1.28)	–
Grade I HT	68	3653	24,102	1.01 (0.76–1.35)	0.10
Grade II HT	19	775	5004	1.44 (0.89–2.32)	0.87
Grade III HT	5	136	869	2.24 (0.92–5.47)	0.42
Treated					
Normal BP	42	1722	11,395	1.14 (0.80–1.62)	0.78
High normal BP	52	1779	11,966	1.28 (0.92–1.78)	1.71
Elevated BP	56	2913	19,104	0.92 (0.67–1.27)	–
Grade I HT	47	2071	13,622	1.05 (0.74–1.48)	0.34
Grade II HT	11	370	2395	1.42 (0.76–2.65)	0.49
Grade III HT	5	92	595	2.50 (1.02–6.15)	0.45
Total	664	38,252	252,127		5.16

PAFs with HR less than 1 are displayed as “–”

Normal BP, SBP < 120 mmHg and DBP < 80 mmHg; High normal BP, 120 ≤ SBP ≤ 129 mmHg and DBP < 80 mmHg; Elevated BP, 130 ≤ SBP ≤ 139 mmHg and/or 80 ≤ DBP ≤ 89 mmHg

Grade I HT, 140 ≤ SBP ≤ 159 mmHg and/or 90 ≤ DBP ≤ 99 mmHg; Grade II HT, 160 ≤ SBP ≤ 179 mmHg and/or 100 ≤ DBP ≤ 109 mmHg; and Grade III HT, 180 mmHg ≤ SBP and/or 110 mmHg ≤ DBP

*BP* blood pressure, *CI* confidence interval, *HR* hazard ratio, *HT* hypertension, *PAF* population-attributable fraction

^a^Adjusted for age, sex, study area, fiscal year of survey participation, season of the survey, smoking status, drinking status, BMI, diabetes treatment, dyslipidemia treatment, history of cardiovascular disease, sodium-to-potassium ratio, educational attainment, marital status, physical activity, depressive symptom, history of cancers

Tables 4 and 5 present the results of the analysis restricted to participants aged ≥40 years with no history of CVD. The PAF for all-cause mortality associated with non-normal BP was 8.00% overall, 9.26% in male, and 5.44% in female.

**Table 4:** Multivariable adjusted hazard ratios for all-cause mortality according to BP classification, hypertension treatment status, and PAF, among participants aged ≥40 years without a history of CVD

	No. of death	No. of participants	Total person-years	HR (95% CI)^a^	HR (95% CI)^b^	HR (95% CI)^c^	PAF (%)
Untreated							
Normal BP	332	14,144	93,060	1.00 (Ref.)	1.00 (Ref.)	1.00 (Ref.)	–
High normal BP	207	7225	47,925	1.02 (0.86–1.21)	1.05 (0.88–1.25)	1.05 (0.88–1.25)	0.58
Elevated BP	302	10,245	66,758	1.02 (0.87–1.19)	1.05 (0.90–1.23)	1.06 (0.90–1.24)	1.00
Grade I HT	194	6069	39,795	0.996 (0.83–1.19)	1.04 (0.87–1.25)	1.06 (0.88–1.26)	0.65
Grade II HT	55	1394	8954	1.27 (0.95–1.69)	1.31 (0.98–1.75)	1.32 (0.99–1.76)	0.78
Grade III HT	20	289	1815	2.23 (1.42–3.51)	2.27 (1.44–3.58)	2.28 (1.45–3.59)	0.66
Treated							
Normal BP	105	2748	18,018	1.05 (0.84–1.31)	1.06 (0.84–1.32)	1.07 (0.86–1.35)	0.40
High normal BP	129	2785	18,554	1.20 (0.98–1.48)	1.22 (0.98–1.50)	1.24 (1.00–1.53)	1.47
Elevated BP	165	4976	32,376	0.93 (0.77–1.13)	0.97 (0.80–1.18)	0.99 (0.81–1.20)	–
Grade I HT	158	3549	23,057	1.18 (0.97–1.42)	1.21 (0.99–1.48)	1.26 (1.03–1.53)	1.92
Grade II HT	28	625	3995	1.26 (0.85–1.85)	1.30 (0.88–1.93)	1.30 (0.88–1.92)	0.38
Grade III HT	7	141	910	1.57 (0.74–3.32)	1.61 (0.76–3.41)	1.68 (0.79–3.56)	0.17
Total	1702	54,190	355,217				8.00

PAFs with HR less than 1 are displayed as “–”

Normal BP, SBP < 120 mmHg and DBP < 80 mmHg; High normal BP, 120 ≤ SBP ≤ 129 mmHg and DBP < 80 mmHg; Elevated BP, 130 ≤ SBP ≤ 139 mmHg and/or 80 ≤ DBP ≤ 89 mmHg

Grade I HT, 140 ≤ SBP ≤ 159 mmHg and/or 90 ≤ DBP ≤ 99 mmHg; Grade II HT, 160 ≤ SBP ≤ 179 mmHg and/or 100 ≤ DBP ≤ 109 mmHg; and Grade III HT, 180 mmHg ≤ SBP and/or 110 mmHg ≤ DBP

*BP* Blood pressure, *CI* confidence interval, *HR* hazard ratio, *HT* hypertension, *PAF* population-attributable fraction

^a^Adjusted for age, sex, study area, fiscal year of survey participation, season of the survey

^b^Adjusted for age, sex, study area, fiscal year of survey participation, season of the survey, smoking status, drinking status, BMI, diabetes treatment, dyslipidemia treatment, sodium-to-potassium ratio

^c^Adjusted for age, sex, study area, fiscal year of survey participation, season of the survey, smoking status, drinking status, BMI, diabetes treatment, dyslipidemia treatment, sodium-to-potassium ratio, educational attainment, marital status, physical activity, depressive symptom, history of cancers

**Table 5:** Multivariable adjusted hazard ratios of all-cause mortality according to BP classification, hypertension treatment status, and PAF, stratified by sex, among participants aged ≥40 years without a history of CVD

	No. of death	No. of participants	Total person-years	Hazard ratio (95% CI)^a^	PAF(%)
Men					
Untreated					
Normal BP	182	3876	25,083	1.00 (Ref.)	–
High normal BP	123	2226	14,482	1.09 (0.87–1.37)	0.94
Elevated BP	202	4244	27,322	1.09 (0.89–1.33)	1.54
Grade I HT	129	2586	16,797	1.06 (0.85–1.34)	0.67
Grade II HT	37	658	4201	1.27 (0.89–1.82)	0.73
Grade III HT	15	157	969	2.30 (1.35–3.92)	0.78
Treated					
Normal BP	67	1182	7653	1.05 (0.79–1.40)	0.29
High normal BP	79	1138	7466	1.17 (0.89–1.54)	1.06
Elevated BP	113	2273	14,639	1.01 (0.79–1.29)	0.10
Grade I HT	114	1606	10,286	1.34 (1.05–1.72)	2.67
Grade II HT	19	286	1797	1.32 (0.81–2.13)	0.43
Grade III HT	3	55	352	1.20 (0.38–3.77)	0.05
Total	1083	20,287	131,047		9.26

	No. of death	No. of participants	Total person-years	Hazard ratio (95% CI)^a^	PAF(%)
Women					
Untreated					
Normal BP	150	10,268	67,977	1.00 (Ref.)	–
High normal BP	84	4999	33,442	0.97 (0.74–1.27)	–
Elevated BP	100	6001	39,436	0.99 (0.77–1.28)	–
Grade I HT	65	3483	22,998	1.01 (0.75–1.36)	0.10
Grade II HT	18	736	4753	1.41 (0.86–2.30)	0.85
Grade III HT	5	132	846	2.32 (0.95–5.67)	0.46
Treated					
Normal BP	38	1566	10,365	1.17 (0.80–1.69)	0.89
High normal BP	50	1647	11,088	1.35 (0.96–1.90)	2.09
Elevated BP	52	2703	17,736	0.94 (0.67–1.31)	–
Grade I HT	44	1943	12,771	1.06 (0.74–1.51)	0.40
Grade II HT	9	339	2198	1.25 (0.63–2.48)	0.29
Grade III HT	4	86	558	2.16 (0.79–5.87)	0.35
Total	619	33,903	224,168		5.44

PAFs with HR less than 1 are displayed as “−”

Normal BP, SBP < 120 mmHg and DBP < 80 mmHg; High normal BP, 120 ≤ SBP ≤ 129 mmHg and DBP < 80 mmHg; Elevated BP, 130 ≤ SBP ≤ 139 mmHg and/or 80 ≤ DBP ≤ 89 mmHg

Grade I HT, 140 ≤ SBP ≤ 159 mmHg and/or 90 ≤ DBP ≤ 99 mmHg; Grade II HT, 160 ≤ SBP ≤ 179 mmHg and/or 100 ≤ DBP ≤ 109 mmHg; and Grade III HT, 180 mmHg ≤ SBP and/or 110 mmHg ≤ DBP

*BP* Blood pressure, *CI* confidence interval, *HR* hazard ratio, *HT* hypertension, *PAF* population-attributable fraction

^a^Adjusted for age, sex, study area, fiscal year of survey participation, season of the survey, smoking status, drinking status, BMI, diabetes treatment, dyslipidemia treatment, sodium-to-potassium ratio, educational attainment, marital status, physical activity, depressive symptom, history of cancers

Supplementary Tables 1, 2 present the results of the analysis after excluding participants who did not respond to questions on hypertension treatment status. The overall PAF for all-cause mortality in the non-normal BP group was 8.35% (13.06% in male and 6.45% in female). When the analysis was restricted to participants aged ≥40 years with no history of CVD, the PAF was 7.46% overall (10.76% in male and 7.20% in female).

## Discussion

This study was a large-scale cohort survey of ~60,000 individuals from the general population, quantitatively evaluating the impact of non-normal BP on all-cause mortality using PAF. The overall PAF for all-cause mortality attributable to non-normal BP was 9.45%, suggesting that ~10% of total deaths could potentially be avoided if all participants were normotensive.

A notable finding in our results is the non-linear relationship between the ascending BP categories and their respective contributions to the PAF. Each category’s contribution is determined not only by its associated hazard ratio (HR) but also by the frequency of deaths within that category. Consequently, the treated Grade I hypertension group, which combines substantial risk with high prevalence, contributed most to the total PAF. This finding highlights the considerable public health burden imposed by moderately elevated but highly prevalent BP categories, suggesting that prevention strategies should target both high-risk individuals and this large, moderate-risk population.

The study also revealed a sex difference in the PAF of all-cause mortality attributable to non-normal BP. The PAF in men (12.25%) was more than twice that in women (5.16%), indicating a greater impact on male mortality. Comparison of HRs across BP categories showed no consistent sex-based differences in individual risk. For instance, in the treated “High normal BP” category, the adjusted HR was 1.11 in men versus 1.28 in women, indicating slightly higher risk in women for this category. Therefore, differences in individual relative risk alone are unlikely to account for the observed sex disparity in PAF. Conversely, a clear sex difference was observed in the distribution of participants across BP categories. The proportion of women with normal BP was 33.5%, compared with 20.0% of men, leaving 80% of the male cohort exposed to some level of hypertension risk versus 66.5% of women. This distribution bias, rather than differences in individual risk, largely explains the higher PAF observed in men.

Past Japanese studies have reported the PAF of all-cause mortality attributable to non-normal BP. A meta-analysis of 13 cohort studies (baseline 1977–1993) reported values of 22.7% for men and 17.9% for women [7], while a nationwide cohort study across nine public health center districts (baseline 1990–1993) reported 17.9% for men [8]. In the present study, a sensitivity analysis restricted participants to those aged ≥40 years with no history of CVD, consistent with these earlier studies. The resulting PAF was 9.26% for men and 5.44% for women (Supplementary Table 2), indicating lower values than previously reported. Several methodological factors may explain this difference. First, the follow-up period in this study (median 6.5 years) was shorter than in previous reports (mean 9.6 years for men and 9.9 years for women in one study [7]; median 11.0 years in the other [8]). Consequently, the full long-term impact of non-normal BP on mortality may not have been entirely captured, potentially contributing to a lower PAF. Second, regional heterogeneity in medical care could influence HRs for a given BP level, as the quality and intensity of management for comorbid cardiovascular risk factors—such as dyslipidemia and diabetes—varies geographically. Third, differences in statistical adjustment likely contributed to the discrepancy. This study controlled for an extensive range of confounding factors, including educational attainment, marital status, physical activity, depressive symptoms, and urinary sodium-to-potassium ratio [19–26], whereas previous studies included fewer covariates, possibly leading to overestimated HRs and PAFs. Generally, more comprehensive adjustment refines risk estimates and can reduce the apparent PAF.

In addition to these methodological factors, the historical context differs substantially. Previous studies had baseline surveys between 1977 and 1993, whereas this study’s baseline was 2013–2016. Over the past two decades, antihypertensive therapy has advanced, with effective, well-tolerated drugs, such as ARBs, becoming widely available [27]. Management of other cardiovascular risks, including dyslipidemia with statins, has also been standardized [28]. These advances may have reduced the impact of a given BP level on mortality, potentially contributing to a lower PAF, although this cannot be directly demonstrated in the current study design.

In the sensitivity analysis limiting participants to those aged ≥40 years without CVD, the overall PAF decreased from 9.45% to 8.00% (Table 4). This decline is likely attributable to the exclusion of high-risk individuals with prior CVD, in whom elevated BP confers a markedly higher risk of recurrence or death. Excluding this group lowered the average HR, resulting in a reduced PAF. These findings underscore the importance of BP management in secondary prevention of CVD, while highlighting that non-normal BP accounts for approximately 8% of deaths even among individuals without CVD history.

A further sensitivity analysis excluded individuals who did not respond regarding hypertension treatment status. In the primary analysis, non-respondents were classified as “untreated.” Excluding them resulted in a slight decrease in overall PAF from 9.44% to 8.35% (Supplementary Table 1). Non-respondents were older (64.6 ± 8.2 years vs. 60.0 ± 11.3 years) and had higher mortality rates (5.8 vs. 4.5 deaths per 1000 person-years), suggesting that their exclusion modestly lowered the PAF. Nevertheless, the difference was limited, confirming that the primary analysis’ treatment of non-respondents did not materially affect the study’s conclusions.

This study has several limitations. First, participation was voluntary, which may have introduced selection bias by producing a relatively healthier study population. Nevertheless, this is unlikely to have substantially affected the observed association between BP categories and all-cause mortality. Second, the study was conducted at two facilities using different mortality follow-up methods. Although some deaths may have been overlooked in the ToMMo data, the extent of potential under-ascertainment remains unknown.

Despite these limitations, this study quantitatively demonstrated, using PAF, that non-normal BP continues to impose a substantial public health burden on all-cause mortality among contemporary Japanese community residents. Overall, ~10% of deaths were attributable to non-normal BP, with the effect notably more pronounced in men, where the contribution to mortality exceeded twice that observed in women. Furthermore, even when restricted to a relatively healthy population without a history of CVD, non-normal BP accounted for 8% of deaths. These findings reaffirm the importance of BP management in primary and secondary prevention.

### Perspective of Asia

Japan is a leading country facing demographic challenges, having entered a “super-aging society” ahead of the rest of the world. This situation in Japan is also a microcosm of the major changes facing the entire Asia region. Rapid demographic change, aging, and rapid urbanization in Asia have been noted to be increasing the burden of CVD [29]. PAF calculated in this study quantitatively demonstrates the impact of non-normal blood pressure on mortality in this context. Therefore, the disease burden quantified in Japan, which has become a super-aging society, provides important insights into the importance of public health measures for Asian countries experiencing similar societal changes in the future.

## Conclusion

In conclusion, non-normal BP remains a major contributor to preventable death in contemporary Japan, accounting for ~10% of all-cause mortality. The public health burden is disproportionately high in men, with the PAF more than twice that observed in women, highlighting the need for sex-specific strategies in BP management. Furthermore, our findings indicate that the greatest portion of this burden arises not from the highest-risk individuals, but from the large population with moderately elevated but highly prevalent BP, such as those with treated Grade I hypertension. These results underscore the importance of implementing population-wide risk-reduction strategies alongside targeted management of high-risk groups.

## Supplementary information

Supplementary Table 1

Supplementary Table 2
